# Strengthening surveillance systems for malaria elimination: a global landscaping of system performance, 2015–2017

**DOI:** 10.1186/s12936-019-2960-2

**Published:** 2019-09-18

**Authors:** Christopher Lourenço, Andrew J. Tatem, Peter M. Atkinson, Justin M. Cohen, Deepa Pindolia, Darlene Bhavnani, Arnaud Le Menach

**Affiliations:** 1 Clinton Health Access Initiative, CHAI, 383 Dorchester Ave, Suite 400, Boston, MA 02127 USA; 2 WorldPop, Department of Geography and Environmental Science, University of Southampton, Southampton, UK; 3 Lancaster Environment Centre, Lancaster University, Lancaster, UK

**Keywords:** Malaria, Elimination, Surveillance, Surveillance system

## Abstract

**Background:**

Surveillance is a core component of an effective system to support malaria elimination. Poor surveillance data will prevent countries from monitoring progress towards elimination and targeting interventions to the last remaining at-risk places. An evaluation of the performance of surveillance systems in 16 countries was conducted to identify key gaps which could be addressed to build effective systems for malaria elimination.

**Methods:**

A standardized surveillance system landscaping was conducted between 2015 and 2017 in collaboration with governmental malaria programmes. Malaria surveillance guidelines from the World Health Organization and other technical bodies were used to identify the characteristics of an optimal surveillance system, against which systems of study countries were compared. Data collection was conducted through review of existing material and datasets, and interviews with key stakeholders, and the outcomes were summarized descriptively. Additionally, the cumulative fraction of incident infections reported through surveillance systems was estimated using surveillance data, government records, survey data, and other scientific sources.

**Results:**

The landscaping identified common gaps across countries related to the lack of surveillance coverage in remote communities or in the private sector, the lack of adequate health information architecture to capture high quality case-based data, poor integration of data from other sources such as intervention information, poor visualization of generated information, and its lack of availability for making programmatic decisions. The median percentage of symptomatic cases captured by the surveillance systems in the 16 countries was estimated to be 37%, mostly driven by the lack of treatment-seeking in the public health sector (64%) or, in countries with large private sectors, the lack of integration of this sector within the surveillance system.

**Conclusions:**

The landscaping analysis undertaken provides a clear framework through which to identify multiple gaps in current malaria surveillance systems. While perfect systems are not required to eliminate malaria, closing the gaps identified will allow countries to deploy resources more efficiently, track progress, and accelerate towards malaria elimination. Since the landscaping undertaken here, several countries have addressed some of the identified gaps by improving coverage of surveillance, integrating case data with other information, and strengthening visualization and use of data.

## Background

Surveillance is the backbone of disease prevention and control [1–3] and is particularly critical to malaria elimination programmes, providing the disease intelligence necessary to target interventions and monitor their effectiveness [4, 5]. Malaria surveillance, defined by the World Health Organization (WHO) Expert Committee on Malaria as “the part of the programme aimed at discovery, investigation and elimination of continuing transmission, the prevention and cure of infections, and final substantiation of claimed eradication” [6], has long been recognized as a key component of malaria elimination [5–7]. Countries that have successfully eliminated malaria have typically relied on a combination of effective passive case detection (PCD) [8–10] and active case detection (ACD) activities [10–13], with staff dedicated to surveillance at all health system levels [11, 14, 15], and integrated response mechanisms [12, 16, 17].

The 2017 WHO Framework for Elimination suggests that achieving malaria elimination requires countries to transition from surveillance systems reporting aggregated case data towards systems that facilitate rapid confirmation, investigation, and reporting of individual cases, and provide enhanced analytic methods to guide decision-making related to anti-malaria interventions [18]. WHO’s Global Technical Strategy for Malaria 2016–2030 (GTS) also describes the need to transform malaria surveillance into a core intervention allowing the identification, tracking, classification and response for all malaria cases to effectively support case management (i.e. providing diagnosis and treatment at health facility or in the community through community health workers or during active surveillance activities such as reactive case detection) and vector control activities (e.g. Indoor Residual Spraying) [19]. This recommendation is supported by several country case studies in Bhutan, Cape Verde, Malaysia, Mauritius, Philippines, Réunion, Sri Lanka, Tunisia, Turkey and Turkmenistan which found that investment in robust, response-driven surveillance systems is critical for malaria elimination [20]. New guidelines for malaria surveillance appeared in early 2018 [21], reinforcing the GTS and Framework for Elimination principle that surveillance should be a primary intervention.

Despite this wealth of experience and guidance, surveillance systems remain inadequate to support elimination targets in many countries [22–24]. In the 2017 World Malaria Report national estimates of malaria case incidence in 32 high-transmission, African countries were derived from parasite prevalence household surveys rather than health information systems given that the quality of surveillance data was considered to be insufficiently robust [25]. Epidemiological indicators vary greatly according to the quality of the data source. For example, in India, estimates of the number of deaths annually range from an average of 1000 deaths reported per year on the National Vector Borne Disease Control Programme website [26] to 15,000 reported in the World Malaria Report [25] to a lower bounds figure of 125,000 in a nationally representative survey [27]. Finally, despite widespread treatment in the private sector in many highly endemic countries, reporting from the private sector was included in only a few countries in the latest World Malaria Report, accounting for less than 2% of all reported malaria cases [25].

Considering the aforementioned surveillance challenges, a standardized surveillance system landscaping was conducted between 2015 and 2016 in 16 countries committed to malaria elimination in collaboration with the governmental malaria programmes to assess the readiness of their surveillance systems to support malaria elimination. The objective was to help countries identify surveillance-related gaps, prioritize solutions, and build systems sufficient for malaria elimination.

## Methods

To achieve this objective, a mixed method approach was used including a) a comparison of each country surveillance system against a set of key criteria associated to an optimal system to identify key gaps in a standardized way, b) a qualitative evaluation through key informant interviews to ensure all potential strengths and weaknesses of the system could be captured, and c) a quantitative approach to measure key gaps along the surveillance pathway.

Existing guidelines about surveillance systems for malaria elimination [18–20, 28] which includes the recent WHO malaria surveillance manual [21], CDC guidelines for evaluating surveillance systems [2, 29], and documented examples of surveillance systems in countries that have successfully eliminated [7, 10, 13, 18, 30–45] were reviewed to identify key elements that should be included in an optimal surveillance system. Current surveillance systems in 16 countries aiming for malaria elimination were then compared against this ideal system. Assessment was conducted in 2015 and 2016 across the Greater Mekong subregion (Cambodia, Laos, Myanmar, and Vietnam), Southern Africa (Botswana, Mozambique, Namibia, South Africa, Swaziland, and Zimbabwe), Hispaniola (Dominican Republic and Haiti), and Central America (Costa Rica, Guatemala, Honduras, and Panama). Each of the countries selected for this review are actively pursuing malaria elimination nationally or sub-nationally, were planning revisions to their surveillance systems at the time of the landscaping, and had adequate documentation outlining the technical and operational aspects of their current systems.

Data collection was conducted, first, through review of existing material and datasets, including patient registers, national surveillance databases, epidemiological surveys such as Demographic Health Surveys (DHS) or Malaria Indicator Surveys (MIS), or previous health facility surveys and, second, through interviews with malaria programme managers, surveillance officers, health facility staff, key stakeholders at different levels of the health system whether central or local, and finally through facility surveys when required and feasible. Data collection was informed by standardized spreadsheet templates to ensure same surveillance system component indicators were reported for each country system.

Each country’s surveillance system was described according to (1) the data collected (which variables, how, by whom, from which sector); (2) the data reported (spatial resolution and format of the data, health information platforms used, data management, validation and protection processes); and (3) the outputs (which outputs were produced, how often, by whom), and whether they were analysed and used for response such as feedback to lower levels of the health system or to guide interventions. A set of key indicators were assessed for each of those steps and each country indicator was qualitatively measured as component “lacking or weak”, “planned but not yet implemented or acceptable”, or “in place or strong” (see Additional file 1). Gaps were then highlighted to identify areas that need to be addressed to bring the system up to elimination standards. Recommendations for addressing observed gaps were then identified and prioritized in terms of public health or financial impact and feasibility of implementation.

To assess system coverage quantitatively, a surveillance waterfall chart was used to illustrate the pathway for an incident (i.e., “new”) symptomatic infection in the community to be reported and eventually informing the malaria programme’s surveillance response. This pathway follows the sequential flow of the surveillance system (i.e. seeking care, receiving diagnosis, attending a facility part of the surveillance system, being reported) to ultimately estimate the cumulative fraction of incident symptomatic infections that were captured and reported through surveillance systems. For each country and region this fraction was evaluated as the product of a cascade of factors including the proportions of: (1) symptomatic malaria cases seeking care, (2) those seeking care receiving a parasitological test, (3) the points of care that are included within the surveillance system (e.g. whether private sector is covered within the surveillance system), and (4) those included points that routinely report into the surveillance system (e.g. within the covered sector). The proportion of symptomatic cases seeking care, and the proportion of those receiving a diagnostic test were evaluated using survey data from DHS or MIS [46–63], World Malaria Reports, and other scientific literature [64–68]. The proportion of points of care included within the surveillance system, and among those the proportion reporting [69–80] were assessed using primary surveillance data and government records such as the list of health facilities from each country. A supplemental table lists the specific references for the quantitative cascade (see Additional file 2). In certain cases where measurements were not available, proxy data were used. For example, the availability of diagnostic testing at the point of care was used as a proxy for the proportion of symptomatic cases receiving a test in Cambodia (using stock data from the 2013 ACTWatch Outlet Survey [46]), in South Africa (using a study on case management practices [68]), and in Mozambique (using a study on the impact of malaria control tools [81]). Also, in Vietnam and Laos, no national surveys were available at the time of the landscaping to determine treatment-seeking behaviour for malaria-related fever, so values from independent studies were averaged [48, 64–66, 80]. The results of quantitative assessments of system coverage were represented regionally with box and whisker plots.

Quantitative data and information about the ideal surveillance system criteria were stored and analysed in a descriptive way in MS Excel. Qualitative information was documented and summarized in MS Word and PowerPoint. The main output was a standardized set of slides outlining the key recommendations to improve the surveillance system, which were prioritized based on their feasibility and impact, and shared and reviewed with each country’s malaria programme and technical partners involved in surveillance activities.

## Results

### Recommendations for ideal surveillance systems

**Fig. 1:**
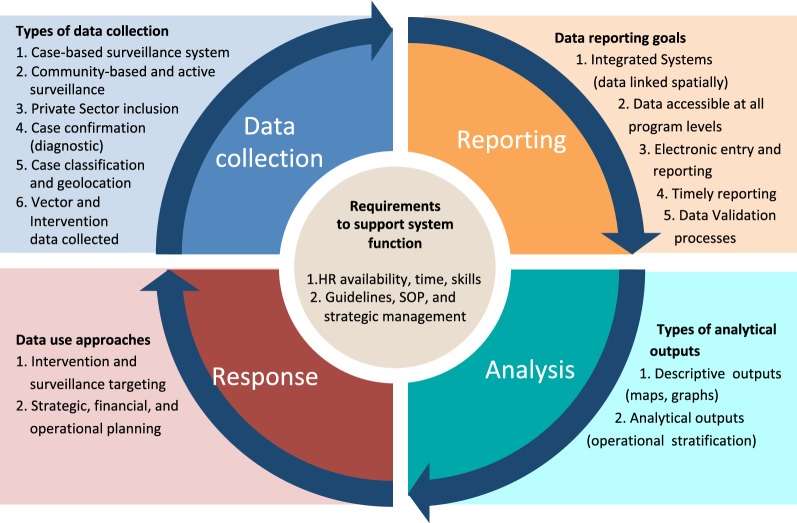
Surveillance loop framework with key indicators for the primary components of data collection, reporting, analysis, and response

The ideal system (Fig. 1) includes data collection at the facility or community level, comprehensive reporting into a health information system, routine analysis and monitoring, and appropriate response, including data-driven planning of malaria elimination strategies, leading iteratively to additional data collection. The framework also includes overarching requirements to support system functioning, such as human resources and strategic management.

The ideal surveillance system should collect data at all levels of the health system and in all sectors including public, private, and others like the military. Indicators should include epidemiological information about key case demographics, diagnosis, geolocation, and classification. Information about vector control interventions, entomological surveillance, commodity and stock management should be collected along with case information, while M&E indicators on performance of the surveillance system itself should also be available. Information should be captured at the lowest spatial resolution possible and linked to the household or village where cases were identified or infection likely occurred.

Data should ideally be reported individually via a case-based surveillance system. To ensure quality, timely and complete data collection, simple and easy-to-understand electronic interfaces should be used that do not require users to be familiar with technology and include built-in validation mechanisms. Validated and standardized malaria information should be secure while accessible at different levels to users with different privileges through user-appropriate dashboards, facilitating the monitoring of temporal and spatial patterns by surveillance-specific staff. The system should be able to permit integrated display and analysis of information from different sources including epidemiological data on case and *foci* investigations as well as data on commodities, interventions, and entomological surveillance.

Data analysis should describe the malaria situation in space and time, identify transmission *foci*, and should support strategic decisions based on operational stratification. The analysis of epidemiological information along with other data such as on entomology and interventions can inform this stratification process and guide the type of interventions chosen for each stratum.

The review and analysis of reported data should facilitate routine monitoring and evaluation of the malaria situation and the performance of the surveillance system and, thus, guide the necessary response. Feedback mechanisms from the central to lower levels of the health system should exist (e.g., through generating automated bulletins or access to tailored dashboards specific for certain operational units within the health system or malaria programme). The system could have pre-defined algorithms triggering alerts (e.g., via SMS or other platforms) and linked response mechanisms that help target active surveillance to the places where surveillance data suggest they are required, or help identify anomalies (e.g., outbreaks). The system should inform the targeting and planning of interventions and facilitate monitoring of their implementation at an appropriate spatial resolution.

The performance of the surveillance system for malaria elimination also relies on a set of cross-cutting factors. Dedicated human resources must be trained and supervised to implement the system, including a focal person at the national malaria control programme as well as surveillance staff at each administrative level, and with refresher training as needed given staff turnover. Clear standard operating procedures (SOPs) will reduce the needs for repeated training and increase the sustainability of the system over time. In addition, strong programme management and leadership, sustainable financing, and potentially some legal policy changes, such as ensuring malaria is a notifiable disease, will create the necessary environment for strong elimination surveillance.

### Assessing surveillance systems in elimination countries

Results of specific country assessments are reported anonymously here to preserve programme sensitivity about sharing country-specific surveillance system details or poor surveillance performance, with results summarized by geographic region. At the time of the review, common gaps in data collection included insufficient surveillance coverage across all relevant geographical areas and health sectors. Three out of 16 countries had an established network of community health workers (CHWs) or active surveillance incorporated and reporting into their surveillance system and three out of 16 included the private sector in their system. In addition, limited data were collected about malaria cases, with five countries classifying cases as local or imported. Ten out of 16 countries reported only parasitologically confirmed (either through microscopy or RDT) malaria cases. One out of 16 countries integrated other types of information such as entomology or vector control.

Six countries had a case-based reporting system with seven others planning to transition from aggregate reporting. One had an integrated system in place with data linked spatially, while others typically had parallel reporting mechanisms for different information from different levels of the system. Most systems relied on paper reporting at local level (14/16) and lacked validation processes to ensure the quality of the reported data (15/16). None of the countries reported having mechanisms for making data easily accessible at lower levels.

Most of the countries produced high quality descriptive outputs of the collected data at the central level (10/16), but these countries provided limited or no feedback to the lower administration levels. Application of surveillance intelligence for monitoring, planning and implementing evidence-based responses was limited and intervention data were often not incorporated back into the surveillance system. Operational stratification was conducted in 12 countries even though it was mostly *ad*-*hoc* and based solely on reported incidence. Data were analysed routinely to guide targeting of interventions in four countries. Three of the 16 countries demonstrated a regular use of surveillance data to inform operational planning. The majority of the countries experienced challenges regarding human resource capacity and expertise (14/16), and there was a need to refine or formulate guidelines or SOPs in 13/16.

### Regional findings: qualitative summaries

At the time of the review, none of the four countries included from the Greater Mekong Subregion (GMS) had systems that could support rapid case-based reporting. One of the countries collected case-based data from endemic areas, but did not report them in a timely manner, with a delay of at least 1 month before data were accessible at the central level. Each country had a system that suffered from lengthy paper-based record keeping and validation processes at each health administration level. No country routinely investigated cases nor conducted case classification. Three of the countries had systems that did not geo-locate cases beyond aggregated health administration units. All four GMS countries produced detailed descriptive outputs of the epidemiological situation. However, these analyses were not routine and were not produced via a standardized interface (such as using dashboards to display key outputs) in three of the four countries. Consequently, system data were not used to inform intervention targeting or programme planning. Finally, at the time of the review, there were no overarching surveillance strategies in each of the countries; no operational guidelines, procedures, or training structures existed specifically to help the programmes direct surveillance system use and subsequent targeting of interventions.

In the Mesoamerica and Hispaniola region, five of the six countries reported case-based data and confirmed all notified cases. However, long delays were often found between case confirmation and case notification. Heavy reliance on paper forms at the local level and transportation of these paper forms often from remote areas to the municipal, departmental or central level for data entry contributed to these long delays. At the time of review, three of the six countries routinely conducted case investigation and classified cases. In two of the six countries, cases were not geo-located. In all countries, other relevant data such as entomological surveillance, indoor residual spraying (IRS) activities, long-lasting insecticide-treated bed net (LLIN) distribution, and active surveillance were collected on paper forms or recorded locally, but not databased or electronically linked to case data. Each of the six countries reviewed reported parallel reporting systems, often due to different health entities or offices housing their own system. Surveillance data, although analysed descriptively at the central level in all countries, were rarely made available to the local level. Finally, there was an overall lack of capacity at the local level to analyse and interpret system data for effective response planning.

In southern Africa, five out of the six countries had national or sub-national elimination goals in place, and all of those five had high case confirmation rates and a case-based surveillance strategy in place. However, two of the five countries were implementing that strategy consistently and at scale, and one had a strong community and active surveillance component with clear case classification and geo-location included in data collection protocols. No country in the region was found to perform well at collecting and/or integrating high quality vector control and entomological data. Data validation and accessibility were the most critical reporting challenges noted for all countries. Five out of the six countries analysed data routinely in time and space with varying degrees of quality. Targeted response was implemented in countries where data collection and reporting was of high quality. As with other regions, human resource challenges were noted across all countries, and four of the countries lacked adequate surveillance guidelines.

### Regional findings: quantitative cascade

**Fig. 2:**
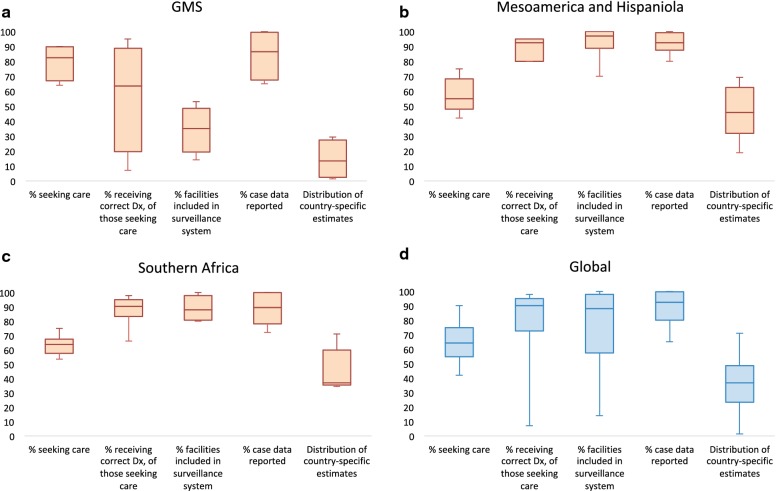
Distribution of values from country assessments for seeking care, receiving diagnosis, facility inclusion, and reporting rates, along with the country-specific total coverage resulting from their product in **a** the GMS (*n *= 4 countries), **b** Mesoamerica and Hispaniola (*n *= 6 countries), **c** Southern Africa (*n *= 6 countries), and **d** Globally (*n *= 16 countries). The box and whisker plots include the median values, minimum and maximum ranges, as well as upper and lower quartiles in the box. In the *x* axis, percentage values of those seeking care are of incident symptomatic malaria infections, the percentage receiving correct diagnosis is the fraction of those seeking care, the percentage of cases from facilities included in the surveillance system is the fraction of those receiving the correct diagnosis, and the percentage of cases reported is the fraction of the cases from facilities included in the surveillance system. The total proportion represents the multiplication of the percentages at each step of the cascade

The proportion of symptomatic cases that were found to seek care, receive a parasitological test, attend a point of care covered by the surveillance system, and be reported correctly were estimated (Fig. 2), and the total fraction of symptomatic cases captured by the passive surveillance system was estimated as their product. The GMS had the smallest estimated proportion of symptomatic cases captured at 13% (quartile range: 2–27). The median proportion of symptomatic cases that sought care was 83% (67–90), 64% (20–89) received a parasitological test, 35% (19–49) attended a point of care included within the surveillance system, and 87% (68–100) were reported. The largest gap in the GMS was the high volume of private sector facilities receiving malaria patients that are not integrated into surveillance systems. In Mesoamerica and Hispaniola, the total proportion of symptomatic cases captured by the passive surveillance system was estimated to be 46% (32–62). The median proportion of symptomatic cases that sought care was lower than the GMS at 55% (48–68), although 93% (80–95) were estimated to receive a parasitological test. 97% (89–100) attended a point of care included within the surveillance system, and 93% (88–99) were reported. In Southern Africa, the total proportion of symptomatic cases captured by the passive surveillance system was estimated to be 37% (35–60). The median proportion of symptomatic cases that sought care was 63% (58–68), 91% (83–95) received a parasitological test, 88% (81–98) attended a point of care included within the surveillance system, and 90% (78–100) were reported.

Globally, the median proportion of symptomatic cases captured by the passive surveillance system was estimated to be 37% (23–49). The proportion that sought care was 64% (55–75), 90% (73–95) received a parasitological test, 88% (57–98) attended a point of care that was covered by the surveillance system, and 93% (80–100) were reported correctly. This analysis identified lack of treatment-seeking in the public health sector as the main bottleneck to the ability of a system to capture cases [median 64% (55–75)]. However, in countries where the private sector accounts for a large share of treatment-seeking behaviour and those facilities are not integrated into the surveillance system (such as those in the GMS), reporting was the main bottleneck.

## Discussion

Surveillance is critical to malaria elimination goals, yet the assessments described here suggest the state of surveillance systems in 2015–2016 was insufficient to support planning and implementing of targeted interventions and to measure progress towards malaria elimination. Overcoming these gaps requires: capture of remote locations within the surveillance system by increasing geographical coverage of access to care and surveillance; design and development of improved architectures that integrate electronic data collection and reporting systems; definition of core data analysis and use for response; and increased capacity to support the deployment of data collection and reporting platforms.

First, programmes should ensure remote at-risk places are captured in the surveillance system to increase geographical coverage of access to care and surveillance. Increasing access to care and surveillance can be achieved through the introduction, expansion and better allocation of CHWs or the implementation of active surveillance activities in targeted areas identified as at risk of transmission (e.g., work sites located in forested areas in the GMS). For example, Sri Lanka’s elimination strategy relied on expanding case detection by mobile malaria clinics (MMCs) targeting pregnant women and military personnel located in remote communities and conflict zones [10, 13]. Another way to expand reporting coverage is through the involvement of the private sector. Population Services International (PSI) supported a public-private mix (PPM) programme in Lao PDR where 55% of enrolled outlets migrated their reporting to PSI’s Android surveillance app, allowing immediate case-based data submissions into the DHIS2-based government health management information system (HMIS) within 24 h [82].

Second, designing and developing improved system architectures that integrate electronic data collection and reporting will improve the quality and availability of data for decision-making. Data collection forms should be simplified to remove information that is not being used or relevant for decision-making while adding key metrics such as travel history to be able to classify malaria cases as local or imported. Electronic data collection (supported by availability of infrastructure and trained human resources) would improve the timeliness of reporting along with higher quality data by including skip-logic and validation processes. For example, when Thailand switched from a monthly, aggregated, paper-based system to electronic collection of malaria data at the facility level in 2016, user acceptability increased, case-based data became available more quickly at multiple levels of the government, the quality of captured data increased, and the overall performance of malaria programme operations improved [83]. Additionally, data from different sources including surveillance, entomological, commodity and intervention information should be integrated (e.g., through the development of a data warehouse) and linked together at some common spatial resolution. This would prevent the development of parallel systems while allowing the analysis of different types of data together. For example, Zambia has used a geographic information system (GIS)-based decision support system, which facilitates the collection and comparison of vector, intervention and epidemiological data in time and space to assess the impact of their interventions and direct limited vector control resources more cost-effectively [84].

Third, assessments highlighted the need to improve data use and data culture among the malaria programmes. Adopting standardized and effective supervision processes can improve data culture, as demonstrated by the 2013 Promoting Malaria Prevention and Treatment (ProMPT) programme in Ghana [85]. This project facilitated supportive supervision and malaria data review meetings, in coordination with local training institutions, which resulted in increased quality and timeliness of district reporting, and which increased the reporting rate of health facilities from 18 to 54% in just 4 months [85]. The USAID-supported MEASURE-PIMA project in Kenya identified data review meetings as the most effective activity to review the quality of available data and improve information use for decision-making [86]. Increasing the usage of surveillance systems for action can be supported by the deployment of user-friendly dashboards that display key indicators relevant to decision-making, accessible and tailored to the needs of different health levels. In 2014, as elimination activities intensified in Bhutan, a spatial decision support system (SDSS) was built that enabled the programme to collect, map, and compare intervention and case data at the household level, which aided in the distribution of LLINs, IRS targeting and for ACD data collection [87].

This review describes landscaping conducted in 16 countries, a subset of the 36 countries committed to elimination [25] and, thus, the results may not be representative of all countries seeking elimination. In addition, within the countries reviewed here, some information was not available because either some programmes lacked strong documentation about the surveillance systems or access to certain data was not possible due to lack of approval or confidentiality. In one country, approval was not granted to review surveillance information from facility level, so the landscaping relied on district and central level data. Also, the information for the cascades in the quantitative component comes from different data sources and as such may limit comparability. More quantitative assessment approaches exist (e.g. the PRISM framework), but require extensive time and resources which constrained their use in this case [88]. Finally, the framework itself was defined qualitatively and the interpretation of some system components may have varied according to certain country contexts. For example, some components such as case classification may be described in guidelines and believed to be of high quality at central level, but there may, in reality, be some challenges to operationalize it at the local level.

Since this landscaping was conducted, most of the countries involved have made meaningful steps towards addressing the gaps identified by these assessments. New surveillance processes such as case based reporting, case investigation and classification have been implemented as pilot or at scale in all the countries. The coverage of surveillance systems has been extended via the introduction or expansion of CHWs in two countries in the GMS, two in Mesoamerica-Hispaniola, and two in Southern Africa. System architectures have been improved through implementation of new electronic, case-based surveillance or revised reporting procedures in three countries in the GMS, three in Mesoamerica-Hispaniola, and four in Southern Africa. Also, two countries in the GMS, two in Mesoamerica-Hispaniola, and four in Southern Africa have been integrating their malaria surveillance systems with entomological surveillance and intervention tracking. Finally, the routine review and use of data collected by surveillance systems has improved in two countries in the GMS, three in Mesoamerica-Hispaniola, and three in Southern Africa through creation of dashboards and/or through initiation of new data review meetings and training. Together, these improvements address several of the critical gaps identified through this review and strengthen regional efforts to eliminate malaria in the near term.

Addressing the barriers to achieving adequate surveillance systems and the use of the landscaping recommendations rely on several approaches. First, ongoing engagement with the programmes before, during, and after the landscaping, and with other key malaria partners was needed to ensure adoption of the landscaping approach and outcome. An ongoing and iterative dissemination of the landscaping results was needed to ensure the recommendations were well received and integrated into strategy and future surveillance strengthening activities and aligned with the programme needs and strategy. Secondly, a strong understanding of programme operations was needed to help prioritize the most impactful and feasible interventions in each country context. Lastly, an understanding of the timing and availability of funding was critical. System improvements should align with programme strategic planning, timing of donor proposal development, and resource allocation to the proposed recommendations. For example, the gaps observed in the GMS country landscaping informed the development of the Regional Artemisinin-resistance Elimination Initiative (RAI2E), a 243 million dollar regional grant to accelerate elimination of *Plasmodium falciparum* malaria in the GMS over a 3-year period (2018–2020), which included a component to strengthen national malaria surveillance systems [89].

## Conclusion

Surveillance is a core intervention to achieve elimination, and countries that have eliminated malaria have established strong information systems and maintained them to prevent the re-establishment of the disease [3, 4, 18, 20, 21]. Assessments in 2015–2016 identified clear gaps in the reach of systems to remote, high-risk areas, reporting from private providers, integration of various data elements, user-friendly visualization of system information, and the routine use of available data to make programmatic decisions. While perfect systems are not required to eliminate, these gaps will make it more challenging to deploy resources in optimal ways, making elimination aspirations more difficult to achieve. In response to these assessments, several countries have made substantial improvements to priority areas of their surveillance systems, including extending them to include additional points of care, shifting from aggregate to case-based reporting, and enhancing the routine use of surveillance data. In future work, this landscaping can inform the development of a standardized toolkit for malaria surveillance assessments. As countries improve malaria surveillance and burden declines, the performance and sustainability of these systems will need to be re-evaluated, and linkages to broader public health surveillance systems should be considered. Spending resources on high priority gaps to increase the quality of information and its routine use by programmes may enable more efficient, targeted programmes that are better equipped to achieve and maintain malaria elimination.

## Supplementary information

**Additional file 1.** Summary table of the surveillance landscaping based on the optimal framework.

**Additional file 2.** Sources for quantitative cascades.

## Data Availability

The datasets generated and/or analysed during the current study are not publicly available due to data protection from national malaria control programmes, but can be available from the corresponding author with the country approval.

## Abbreviations

ACD: active case detection
CHW: community health worker
DHS: Demographic Health Survey
GIS: geographic information system
GMS: Greater Mekong Subregion
GTS: Global Technical Strategy for Malaria 2016–2030
HMIS: health management information system
IRS: indoor residual spraying
LLIN: long-lasting insecticide-treated bed net
MIS: Malaria Indicator Survey
MMC: mobile malaria clinic
PAHO: Pan-America Health Organization
PCD: passive case detection
PPM: public–private mix
ProMPT: Promoting Malaria Prevention and Treatment
PSI: Population Services International
SDSS: spatial decision support system
SOP: standard operating procedure
UCSF: University of California—San Francisco
WHO: World Health Organization
