# Supplementary material for: Strengthening surveillance systems for malaria elimination: a global landscaping of system performance, 2015–2017
Source: Malar J. 2019 Sep 18;18:315. doi: 10.1186/s12936-019-2960-2 (PMC6751607; doi:10.1186/s12936-019-2960-2)

Additional File 1.

Summary table of the surveillance landscaping based on the optimal framework.
Each country in the landscaping was reviewed against the surveillance components outlined in each main stage; data collection, reporting, analysis, response, and operations. Each country component was qualitatively measured and marked as “lacking or weak”, “planned but not yet implemented or acceptable”, or “in place or strong”. Country names were removed for confidentially.

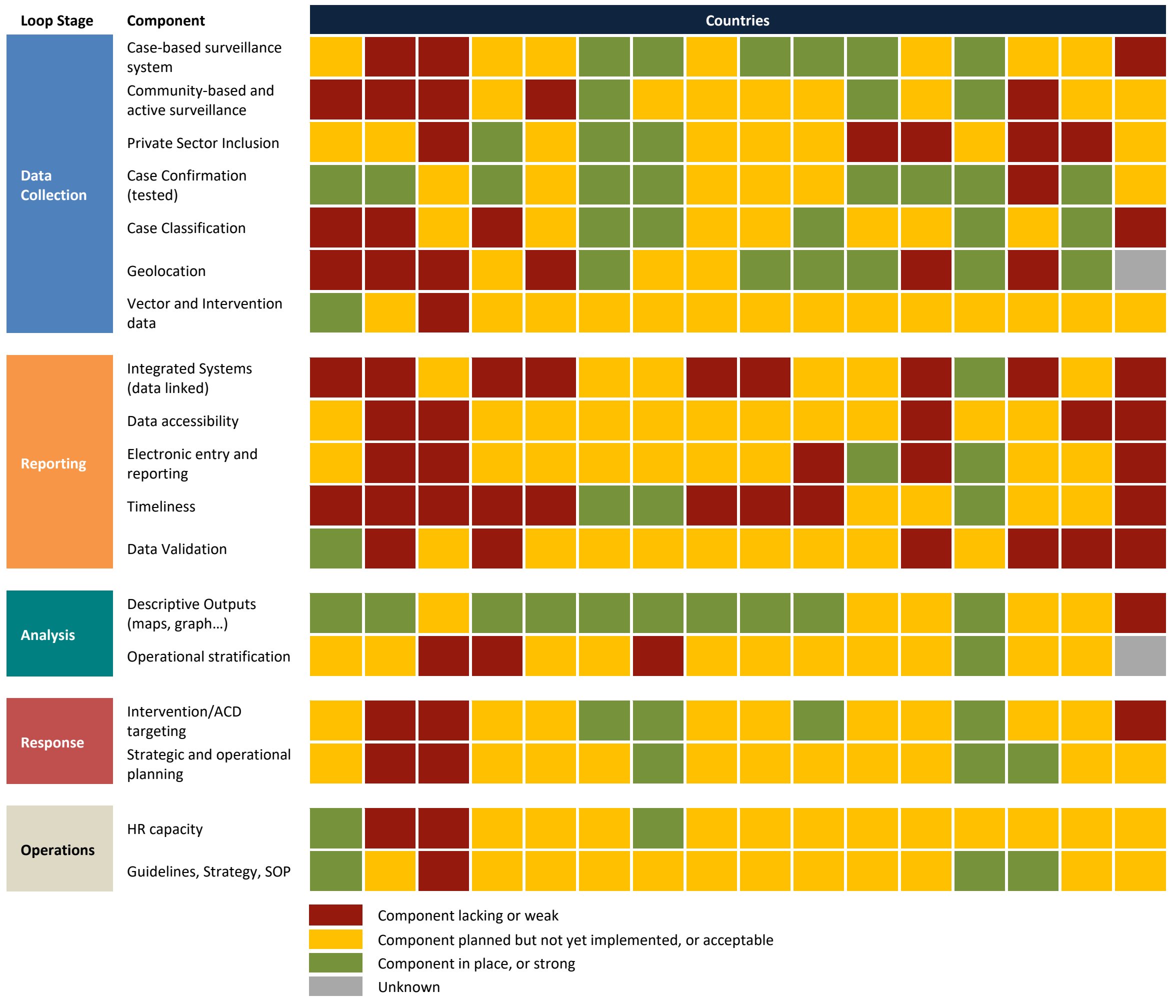

Supplement: Supplementary file 1 — Additional file 1. Summary table of the surveillance landscaping based on the optimal framework. [file 12936_2019_2960_MOESM1_ESM.pdf]
