# Supplementary material for: Strengthening surveillance systems for malaria elimination: a global landscaping of system performance, 2015–2017
Source: Malar J. 2019 Sep 18;18:315. doi: 10.1186/s12936-019-2960-2 (PMC6751607; doi:10.1186/s12936-019-2960-2)
Supplement: Supplementary file 2 — Additional file 2. Sources for quantitative cascades. [file 12936_2019_2960_MOESM2_ESM.pdf]

Additional File 2. Sources for Quantitative Cascades

	Seeking Care	Diagnosis	Facility Participation	Reporting
**Vietnam**	University of California, San Francisco. The Private Sector's Role in Malaria Surveillance. December 2014.	National Institute of Malariology, Parasitology and Entomology (NIMPE) malaria database, and Annual Program Review, 2015	NIMPE paper-based reporting system data	NIMPE Annual Program Review, 2015
**Cambodia**	Cambodia Malaria Survey, 2013	ACTWatch Outlet Survey, 2013	Malaria Information System (MIS), data by health center level, 2014	Health Information System (HIS), data be operational district (OD), 2014
**Laos**	Nonaka D, Vongseththa K, Kobayashi J, Bounyadeth S, Kano S, Phompida S, Jimba M: Public and private sector treatment of malaria in Lao PDR. Acta Tropica 2009, 112:283–287.	Centre for Malaria Parasitology and Entomology (CMPE), The Global Fund to Fight AIDS, Tuberculosis and Malaria (GFATM) Revised Progress Update and Disbursement Request (PUDR), 2014	MIS, data by district, 2014	MIS, 2015
**Myanmar**	ACTWatch, 2014	ACTWatch, 2014	Malaria Consortium (MC) Surveillance Assessment, 2013	Malaria Consortium (MC) Surveillance Assessment, 2013
**Botswana**	National Malaria Control Program (NMCP) consultation, 2015 (unpublished data)	HIS data, 2014	Integrated Disease Surveillance and Response (IDSR) Unit data, 2014	HIS data, 2014
**Mozambique**	Demographic and Health Survey (DHS), 2011	Centro de Investigação em Saúde de Manhiça (CISM) RDT study in 4 provinces, 2013	Boletim Epidemiologico Semanal (bulletin for notifiable diseases, BES), 2014	Annual summary file from BES with cases and intervention data from 2011 through 2014
**Namibia**	DHS, 2013	Weekly Surveillance System (WSS), 2014	DHS, 2013	NMCP consultation, 2015 (unpublished data)
**South Africa**	DHS, 2003	Frean et al. 2013, SAMJ - Case management of malaria: Diagnosis	DHS, 2003	World Malaria Report, 2015
**Eswatini**	Malaria Indicator Survey, 2010	NMCP seasonal data, 2014-2015	NMCP seasonal data, 2014-2015	NMCP seasonal data, 2014-2015
**Zimbabwe**	MIS, 2012	PMI Malaria Operational Plan, 2015 (using DHIS2 data)	NMCP presentation at Elimination 8 meeting, July 2015	World Malaria Report, 2014
**Haiti**	Haiti Mortality, Morbidity and Service Utilization Survey, 2012	Evaluation of Malaria and Antimalarial Services in Haiti's Health Facilities, 2014-2015	Evaluation of Malaria and Antimalarial Services in Haiti's Health Facilities, 2014-2015	(Haïti Système d'Information Sanitaire) HSIS data, 2014
**Dominican Republic**	DHS, 2013	NMCP consultation, 2015 (unpublished data)	NMCP consultation, 2015 (unpublished data)	Ministry of Health (MoH) case data, 2008-2015
**Guatemala**	DHS, 2014-2015	NMCP consultation, 2015 (unpublished data)	NMCP consultation, 2015 (unpublished data)	NMCP consultation, 2015 (unpublished data)
**Honduras**	DHS, 2011-2012	Knowledge, Attitude and Practices (KAP) Survey, 2011	Knowledge, Attitude and Practices (KAP) Survey, 2011	NMCP consultation, 2015 (unpublished data)
**Costa Rica**	MinSa publicación 2005 - "La inmigración en Costa Rica: Dinamicas, Desarrollo, y Desafíos"	Ministerio de Salud (MinSa) consultation, 2015	Ministerio de Salud (MinSa) consultation, 2015	Ministerio de Salud (MinSa) consultation, 2015
**Panama**	Ministry of Health, Epi Info Case Reports, 2014	Clinton Health Access Initiative (CHAI) Health Facility Survey, 2015	Clinton Health Access Initiative (CHAI) Health Facility Survey, 2015	Clinton Health Access Initiative (CHAI) Health Facility Survey, 2015
